# Increasing Contraceptive Use Among Young Married Couples in Bihar, India: Evidence From a Decade of Implementation of the PRACHAR Project

**DOI:** 10.9745/GHSP-D-17-00440

**Published:** 2018-06-27

**Authors:** Laura Subramanian, Callie Simon, Elkan E. Daniel

**Affiliations:** a Pathfinder International, Watertown, MA, USA. Now with Ariadne Labs, Boston, MA, USA.; b Pathfinder International, Washington, DC, USA. Now with Save the Children, Seattle, WA, USA.; c Pathfinder International, New Delhi, India. Now with Swasti Health Catalyst, Bangalore, India.

## Abstract

**Background:**

Evidence on effective behavior change programming for sexual and reproductive health among married youth aged 15–24 in developing countries is lacking. To address this gap, we examined monitoring, evaluation, and special study data from the Promoting Change in Reproductive Behavior of Adolescents (PRACHAR) Project, which was implemented between 2001 and 2012 in Bihar, India, over 3 phases using 2 different implementation models (NGO- and government-led).

**Methods:**

We conducted a synthesis of evidence from multiple PRACHAR studies to identify key findings on intervention effectiveness, scalability, and sustained effects on behaviors. Data were triangulated from quantitative population-based quasi-experimental evaluations from each project phase; the project's performance monitoring database; and qualitative studies with beneficiaries.

**Results:**

PRACHAR's original comprehensive NGO-led model, which included behavior change elements and multiple overlapping communication channels (including home visits and small-group meetings), increased contraceptive use among young married couples, and these outcomes were sustained 4–8 years after project interventions ended. Several program elements were critical to the effectiveness of PRACHAR, including use of a socioecological intervention model with emphasis on behavior change efforts; use of a gender-synchronized approach that engages both male and female partners; and intensity of interventions calibrated to different moments in the life cycle of adolescents and youth. While the hybrid government-NGO model of PRACHAR implementation reached greater scale than the original NGO-led model, comparison of results suggests trade-offs in effectiveness of interventions and sustained impacts.

**Conclusions:**

A decade of learning from the PRACHAR Project in Bihar, India, finds that comprehensive programming with gender-synchronized interventions tailored to specific life stages and aimed at different levels of the socioecological model can effectively increase contraceptive use among married young people in a conservative context. Shifting from a more intensive NGO-led model to less intensive government implementation enhances scalability but may have diminished impact on reach and long-lasting effectiveness.

## Abstract (precis)

Critical program elements to improving voluntary contraceptive use among married youth included: (1) use of a socioecological intervention model of behavior change; (2) engaging both women and men; and (3) calibrating interventions to different moments in the life cycle of adolescents and youth. Trade-offs between intensive NGO-led models and less intensive government-led models occurred in effectiveness, scale of interventions, and sustained behavior changes.

## INTRODUCTION

In many low- and middle-income countries, early marriage followed by early and closely spaced births results in elevated risk for maternal and infant morbidity and mortality and limited opportunities for educational and economic advancement among young married women.^1^^–^^3^ By age 18, 28% of young women living in developing regions are married or in union, and 90% of the approximately 12 million annual adolescent births in developing regions occur in the context of marriage.^4^^,^^5^ Early and rapid repeat pregnancies and births among young married women (under age 24) are driven by a number of factors, including gendered social norms that require women to demonstrate fertility to prove their value, young women's lack of agency to seek health care, and limited access to contraceptive information and a full range of methods.^3^^,^^6^^,^^7^

In response to persistently low use of contraception and high rates of early and rapid repeat childbearing among young married women, there is a growing call to address the drivers of low contraceptive use and to increase young married couples' access to contraception.^7^^–^^9^ Doing so would address critical unmet need for family planning and contribute to achievement of national and global goals and priorities, such as Family Planning 2020 (FP2020) and the Sustainable Development Goals.^10^^,^^11^ Some efforts have been made to increase contraceptive use among married adolescents and youth and to prevent rapid repeat pregnancies, and recent papers have synthesized the primary strategies used in these programs.^7^^,^^8^^,^^12^ However, there remains little published evidence from low- and middle-income countries to inform critical program design decisions related to intervention intensity and duration, effective combinations of interventions, and scale up. This makes it critical to learn from the few rigorously documented and evaluated projects that have worked with married young women and their partners to address the social and behavioral constraints to contraceptive use. The Promoting Change in Reproductive Behavior of Adolescents (PRACHAR) Project, implemented in Bihar, India, has amassed a wealth of monitoring and evaluation data on its interventions with young married couples, much of which is unpublished. By synthesizing these data and implementation experiences from more than a decade of PRACHAR implementation, this article seeks to contribute to the growing body of evidence around behavior change approaches for contraceptive use by married youth.

This article seeks to contribute to the growing body of evidence around behavior change approaches for contraceptive use by married youth.

## PROGRAM DESCRIPTION

The PRACHAR Project was designed and led by Pathfinder International and implemented in Bihar, India, from 2001 through 2012. At the time PRACHAR began, Bihar had few programs to address the contraceptive needs of the population, including adolescents and youth. The modern contraceptive prevalence rate was low (22%) for all women of reproductive age, with almost no contraceptive use among married adolescents aged 15–19 (1%) and young women aged 20–24 (5%).^13^ Bihar also had the highest prevalence of early marriage in India, with 84% of young women married by age 18.^13^ To address this situation, PRACHAR aimed to delay the age at first birth by delaying the age at marriage and increasing voluntary contraceptive use among young nulliparous married women, and to space second and subsequent births by at least 3 years among young married women in Bihar.

PRACHAR was designed using a life-stage tailored social and behavior change approach, based on the socioecological framework.^14^ PRACHAR interventions targeted individuals (young men and women ages 12–24), their family members and other gatekeepers (husbands, fathers, mothers, and mothers-in-law), community members (religious leaders, community elders), and the health service delivery system. Young women and men in PRACHAR areas could have been exposed to PRACHAR at multiple life stages (before marriage, as newlyweds, and at different parities). For unmarried adolescents, PRACHAR conducted training on sexual and reproductive health and life skills with age-appropriate content for 12–14 and 15–19 age groups, delivered separately to males and females. For newlywed couples, PRACHAR hosted “newlywed ceremonies” that combined education and entertainment. For married young women with up to 2 children, female lay health workers (called “change agents”) conducted home visits and group meetings to counsel and refer women for services at planned intervals timed with life events such as marriage, pregnancy, and newly parenting a child. Male change agents reached husbands of young women through regular small-group meetings, which included dialogue and discussion on sexual and reproductive health and gender. Mothers-in-law were reached with home visits and small groups, and the wider community was engaged through community meetings, street theater performances, wall paintings, puppet shows, and information, education and communication (IEC) materials. Government and private-sector contraceptive services were mapped and received small enhancements (e.g., training) from PRACHAR, with referrals to these services made by the change agents. All activities were mutually reinforcing and used dialogic and narrative content to promote reflection and dialogue that aimed to change attitudes and behaviors related to early marriage, immediate childbearing, birth spacing, and contraceptive use. (See the Supplement for a detailed description of how PRACHAR activities were implemented.)

**FIGURE 1:**
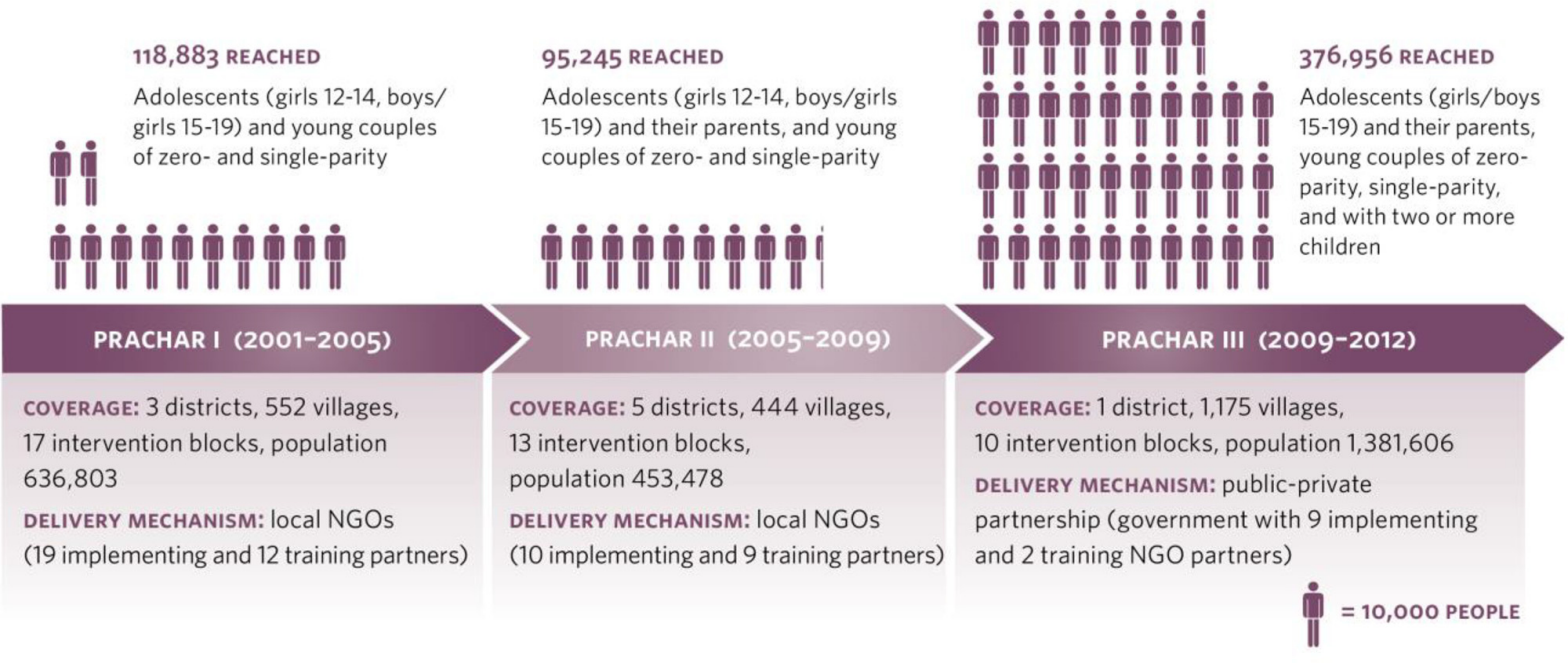
PRACHAR Phases, Intervention Delivery Mechanisms, and Coverage Abbreviation: PRACHAR, Promoting Change in Reproductive Behavior of Adolescents.

PRACHAR was implemented in 3 phases with different coverage levels, intervention combinations, and durations (Figure 1 and Table). Phase I was implemented for 3 years in Nalanda, Nawada, and Patna districts of Bihar, using a comprehensive model including all the interventions described above. Phase II was implemented in 5 districts: the original 3 districts plus Gaya and Shikhpura. Phase II included different intervention arms to compare the effectiveness of the comprehensive PRACHAR model over different durations (2 and 5 years) with 3 “single intervention” models. The “single-intervention” models were: trained couples only (where young married couples were trained to provide reproductive health information), home visits only, and volunteers only (where community resource people were available for reproductive health questions and dialogue without a formal home visit or group meeting structure). All the arms in Phase II included community-level enabling environment activities. Phases I and II were implemented through local NGOs with referrals to government and private-sector service delivery sites. Phase III was implemented for 3 years in Gaya district and aimed to test a streamlined government-NGO model that had greater potential for scale up than the Phase I and II models. Government community-based health workers (Accredited Social Health Activists [ASHAs]) assumed the role of female change agents conducting home visits. Local NGOs maintained responsibility for male engagement and training unmarried adolescents. Engagement with husbands, gatekeepers, and the broader community was diminished. The Phase I, II, and III districts were chosen because they were representative of Bihar from a demographic perspective (the majority of the population was of low socioeconomic status and belonged to the Scheduled and Backward castes) and were within 2–4 hours of the city of Patna (the state capital) by car or bus.

**TABLE.** PRACHAR Activities, by Project Phase

PRACHAR Strategies and Interventions	Target Level	Implementer(s)	Phase I	Phase II					Phase III
			Comprehensive3-yearmodel	Comprehensive 5-yearmodel (continued for 2years beyond Phase I)	Comprehensive2-year model	Homevisitsonly	Volunteersonly	Trainedcouplesonly	2-yearmodel withASHAs
**Interventions with adolescents, young couples, key influencers, and communities to increase support for RH practices and use of services**									
Small-group education on RH with adolescent girls (aged 12–14 years), and unmarried girls and boys aged 15–19 years	Individual	Trainers from NGO training partner	X	X	X	–	–	–	X
Counseling on RH/FP and referrals to FP services through regularly scheduled home visits to married young women with no children, married young women with first pregnancy, married postpartum young women who delivered their first child, married young women with 1 child	Individual	Phase I and II: Female Change Agent Phase III: ASHA	X	X	X	X	–	–	X
Home visits to women and men (whenever possible, without a regular schedule) and referral by female and male volunteers (rather than paid change agents), respectively	Individual	Male and female community volunteers	–	–	–	–	X	–	–
Outreach to young couples by other trained young couples (rather than change agents, peer–to-peer outreach approach)	Individual, young couples	Male and female members of young couples	–	–	–	–	–	X	–
Newlywed couple ceremony/infotainment parties	Young couples, Group	NGO training partner	X	X	–	–	–	–	–
Small-group discussion and dialogue on RH and contraception, and referrals to health services, separately to young married women and married men	Group	Phase I and II: Female Change Agent, Male CommunicatorPhase III: ASHA, Male Communicator	X	X	X	–	–	–	X
Orientation and training of community leaders and influencers on RH for young people	Community	NGO intervention partner	X	X	X	–	–	–	–
Group meetings and infotainment programs for mothers and fathers of young married men (the mothers-in-law and fathers-in-law of young married women)	Community	NGO intervention partner	X	X	X	X	X	X	–
Street theater performances	Community	NGO intervention partner	X	X	X	–	–	–	–
Wall paintings	Community	NGO intervention partner	X	X	X	X	X	X	X
**Improving access to RH services**									
Support to monthly MCH clinics by providing government ANMs with training and support, essential instruments, and recordkeeping tools.	Community	Phase I and II: NGO intervention partner, Anganwadi Worker, ANM (Government)	X	X	X	X	X	X	–
Training of rural health practitioners on RH and FP issues	Community	NGO training partner	X	X	X	X	X	X	–
Training of TBAs on safe delivery, counseling on postpartum contraceptives, and referral of pregnant women with complications	Community	NGO training partner	X	X	X	X	X	X	–
Training of chemist outlets and village convenience shops on FP and connecting them with social marketing agencies to encourage regular stocks of condoms and pills	Community	NGO intervention partner	X	X	X	X	X	X	–

Abbreviations: ANM, auxiliary nurse-midwife; AHSA, accredited health social activist; FP, family planning; MCH, maternal and child health; PRACHAR, Promoting Change in Reproductive Behavior of Adolescents; RH, reproductive health; TBA, traditional birth attendant.

The PRACHAR Project was implemented in 3 phases between 2001 and 2012 with different coverage levels, intervention combinations, and durations.

## METHODS

From 2014 to 15, Pathfinder International staff conducted a review of existing PRACHAR Phases I, II, and III evaluation reports, special studies, presentations, and project monitoring data to synthesize evidence related to the following research question: “What is the evidence from PRACHAR around programming to increase contraceptive use among young married women and men?”. The studies we reviewed are briefly described here, and their methodologies are described in the source files referenced. First, we reviewed reports of quasi-experimental studies conducted for each project phase, i.e. population-based surveys among young married women and men in intervention and control areas at baseline and endline.^15^^–^^17^ In addition, we reviewed reports of special studies conducted to further assess effectiveness of interventions. These included the Adolescent Follow-up Survey with youth in PRACHAR Phase I intervention areas who participated in adolescent trainings and other enabling environment interventions, as well as a comparable control group; a qualitative study on gender norms, attitudes, and practices related to sexual and reproductive health outcomes in PRACHAR intervention and comparison areas; a survey conducted by the Population Council to evaluate the effectiveness of Phase III in building ASHAs' capacity to offer reproductive health services; and a population-based survey conducted by the Population Council to evaluate if the reproductive health outcomes observed in PRACHAR Phases I and II were sustained among new cohorts of women 5–8 years after PRACHAR ended.^18^^–^^21^ We also reviewed analyses of individual-level routine project monitoring data on contraceptive uptake among young married beneficiaries.

Following the review of existing PRACHAR evaluation and monitoring reports, we identified key gaps in knowledge that would be important to address for program design purposes and conducted secondary analyses of PRACHAR evaluation data to answer specific questions around effectiveness, intensity, and duration of program interventions. We generated bivariate frequency distributions and conducted multivariate logistic regressions with contraceptive use as the outcome variable and a variety of independent variables (demographic characteristics such as age, education, wealth index, and parity; exposure to PRACHAR interventions).

The findings from our review and the secondary analysis were categorized according to key program learning themes as described in the Results below.

## RESULTS

Through our review of the PRACHAR evaluation studies, special studies, and our secondary analysis, we identified evidence around 4 key themes: (1) project effectiveness in achieving attitudinal and behavioral outcomes; (2) effectiveness of selected program components and the intensity required to produce effects; (3) scalability and effectiveness at scale; and (4) sustained project impact.

### PRACHAR's Effectiveness in Achieving Attitudinal and Behavioral Outcomes

**FIGURE 2:**
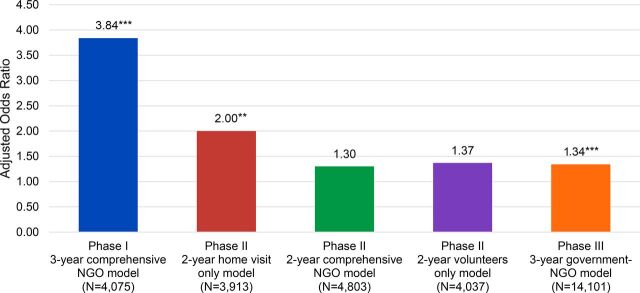
Adjusted Odds Ratios for Current Contraceptive Use Among Young Married Women Aged 15–24 (Phase I and II) and Aged 15–34 (Phase III) in PRACHAR Intervention Models Reference groups are comparison areas where PRACHAR was not implemented. Adjusted odds ratios are from multivariate logistic regressions comparing baseline-endline changes in intervention and comparison areas for each PRACHAR model. These adjusted odds ratios are from different studies/designs so direct comparison should be taken with limitations. *P<.05; **P<.01; ***P<.001. Abbreviation: PRACHAR, Promoting Change in Reproductive Behavior of Adolescents.

The results of our analyses indicate that comprehensive intervention models of longer duration are most effective in increasing contraceptive use among married youth. The 3-year comprehensive NGO-led PRACHAR model in Phase I, which included behavior change elements and multiple overlapping communication channels, had the greatest magnitude of effect on contraceptive use. As published in Daniel et al. (2008), the odds of current contraceptive use increased nearly 4 times as much from baseline to endline among young married women in Phase I intervention areas than among those in comparison areas (adjusted odds ratio [aOR]=3.84; *P*<.001, adjusted for age, education, caste, and parity) (Figure 2).^15^ The adjusted effect size for Phase I is larger than that of PRACHAR's 2-year “single-intervention” models in Phase II (i.e., the 2-year “home visit only” model with an aOR of 2.00, *P*<.01, adjusted for age, parity, education, caste, and standard of living index; the 2-year comprehensive model [not statistically significant]; and the 2-year “volunteers only” model [also not statistically significant]),^16^ as well as the Phase III government-NGO model of similar duration (aOR=1.34; *P*<.001, adjusted for age, education, and caste).^17^ The lack of significant effect on contraceptive use in the comprehensive 2-year model (aOR=1.30) offers suggestive evidence of a minimum duration of comprehensive interventions required to achieve effects in this context.

Comprehensive intervention models of longer duration are most effective in increasing contraceptive use among married youth.

**FIGURE 3:**
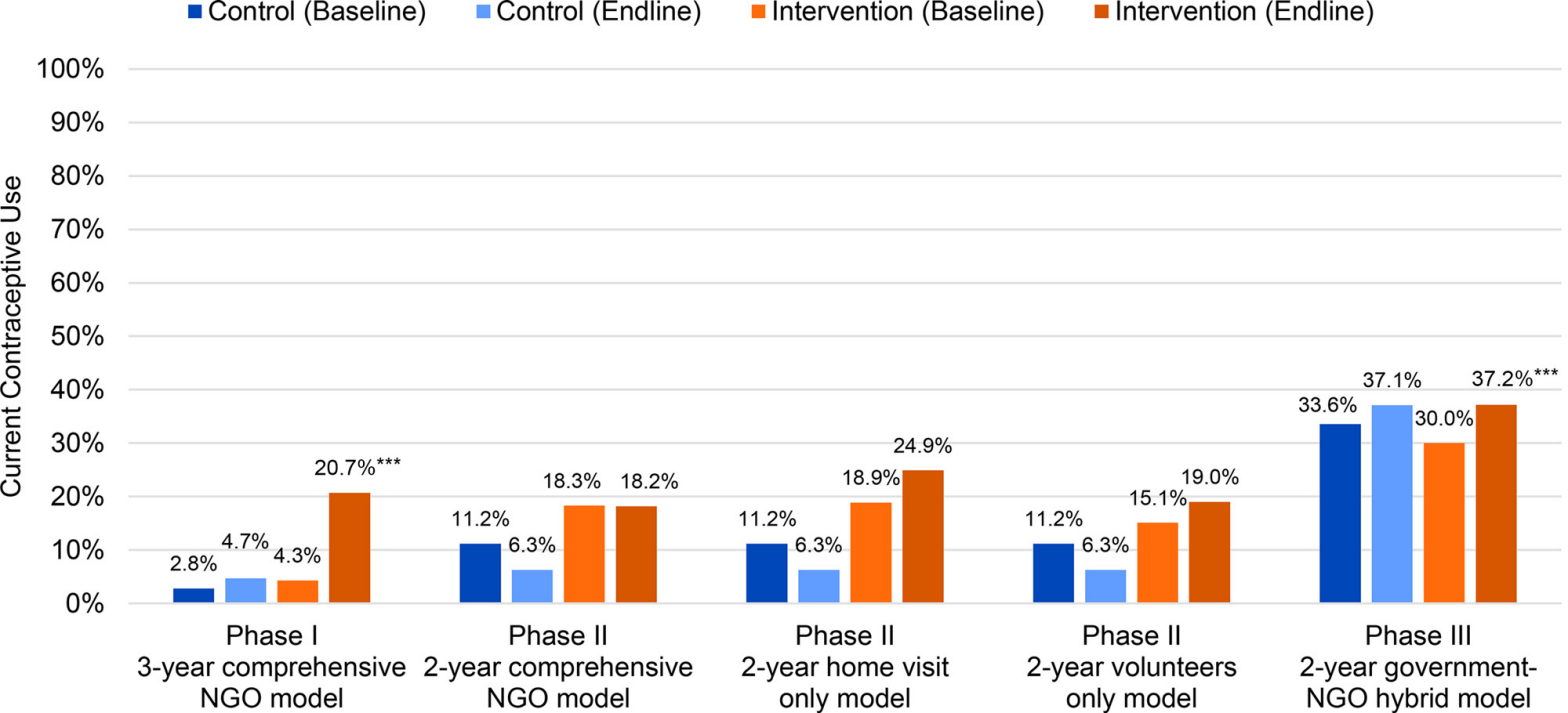
Current Contraceptive Use Among Married Women Aged 15–24 (Phases I and II) and Aged 15–34 (Phase III) in PRACHAR Intervention Models *P<.05; **P<.01; ***P<.001. Abbreviation: PRACHAR, Promoting Change in Reproductive Behavior of Adolescents.

Bivariate findings on current contraceptive prevalence rate from all 3 PRACHAR Phases also show the greatest baseline-endline increase in the 3-year Phase I model, from 4% to 21% in intervention areas (Figure 3).^15^^–^^17^ While the contraceptive prevalence rate findings from Phases I and II are not directly comparable with Phase III due to different study populations (ages 15–24 in the former vs. ages 15–34 in the latter), the relative baseline-endline increase is still greatest in the 3-year Phase I model.

**FIGURE 4:**
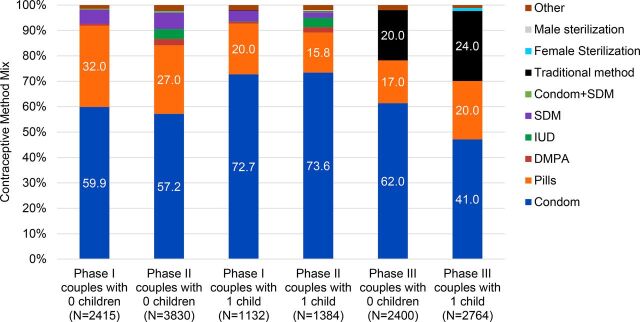
Contraceptive Method Mix Among Young Married Contraceptive Users Aged 15–24 in the PRACHAR 5-Year Comprehensive Model (Phases I and II), and Aged 15–34 in Phase III Intervention Areas (Baseline + Endline) Abbreviation: PRACHAR, Promoting Change in Reproductive Behavior of Adolescents.

Contraceptive method mix remained relatively consistent in Phases I and II, reflecting the limited availability of several methods in Bihar at that time, particularly for young married women. Condoms and pills were the most commonly used contraceptive methods among young married women aged 15–24 of zero and single parity in the intervention and comparison areas. Condoms ranged from 62% to 85% of the method mix, and pills from 11% to 27% of the method mix. Use of IUDs was negligible (less than 1%), and there was virtually no use of female or male sterilization as expected with a young population (Figure 4). In Phase III, contraceptive method mix among women aged 15–34 of zero or single parity remained heavily focused on short-acting methods (condoms and pills), with minimal use of female sterilization and some use of traditional methods.^17^

### Effectiveness of Selected Program Components and the Intensity Required to Produce Effects

Phase I evidence also sheds light on the relative contributions of specific PRACHAR interventions, as well as the intervention timing and intensity required to influence contraceptive use. Home visits by NGO change agents conducting interpersonal communication were found to be effective in increasing contraceptive use among young married women when implemented in tandem with community-level activities that aimed to change attitudes and behaviors. In the Phase I comprehensive model, young married women in PRACHAR intervention areas who were exposed to home visits had 2 times higher odds of currently using contraception than those who did not receive home visits (aOR=2.30; *P*<.001, adjusted for education, caste, and standard of living index). Among the Phase II “single-intervention” models, the home visit model (plus service linkages and community-level interventions) had the highest magnitude of effect for current contraceptive use (aOR=2.00 as noted above; *P*<.01).^16^ No effect was seen in the other “single-intervention” 2-year models. Additionally, bivariate analyses showed that couples reached with both adolescent training and home visits in Phase I had higher rates of ever using contraception than couples reached with only one of these interventions, suggesting a multiplicative effect of these interventions. There is also some evidence of effectiveness of small-group meetings. Phase I and II data show that married women in PRACHAR intervention areas who were exposed to group meetings (and potentially other interventions as well) had 3 times higher odds of currently using contraception than those not exposed (aOR=3.16; *P*<.001, adjusted for education, caste, and standard of living index).

Home visits by NGO change agents, when implemented in tandem with community-level activities, were found to be effective in increasing contraceptive use among young married women.

**FIGURE 5:**
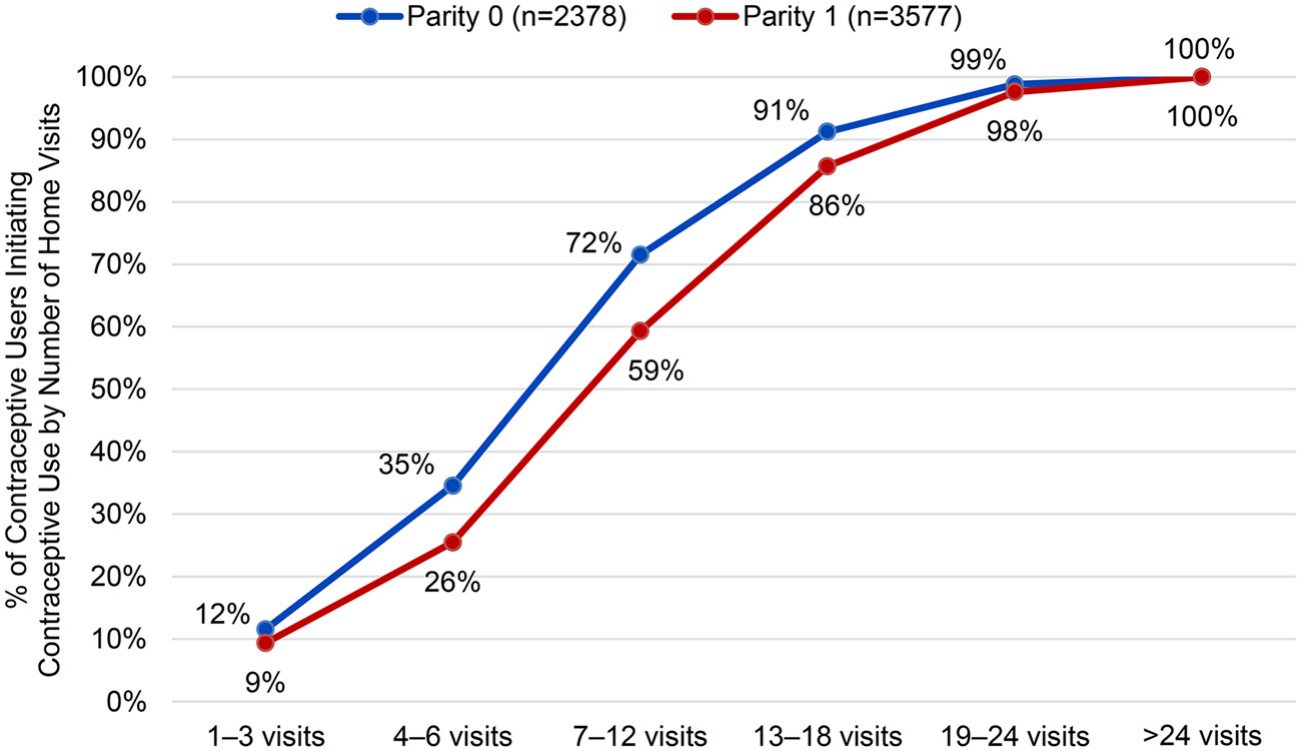
Number of Home Visits Required for Contraceptive Initiation Among Young Married Contraceptive Users Aged 15–24 in PRACHAR Phase I, by Parity Abbreviation: PRACHAR, Promoting Change in Reproductive Behavior of Adolescents.

PRACHAR Phase I monitoring data show that intensity and timing of home visits matter. Women reached with home visits at multiple life cycle stages (newlywed, before pregnancy, during pregnancy, and after first birth) had the highest ever use of contraception after their first birth and initiated contraception more quickly compared with women reached at fewer life cycle stages. In addition, a relationship was observed between the number of home visits and ever use of contraception among young married women, with 7 to 12 visits as the “tipping point” where more than half of contraceptive users had initiated use (Figure 5). While multiple factors affect initiation of contraception, these data suggest that among young women who eventually used contraception, repeated home visits were required to stimulate contraceptive initiation.

We also found that contraceptive initiation earlier in life is correlated with future contraceptive use. Results from the Adolescent Follow-up Survey show that married young women with 1 or more children (in both intervention and comparison areas) who had used contraception before their first birth had nearly 14 times higher odds of using contraception after their first birth compared with women who had not previously used contraception (aOR=13.70; *P*<.001).^21^ This further underscores the importance of reaching young married women to promote contraceptive initiation early in their reproductive life.

Contraceptive initiation earlier in life is correlated with future contraceptive use.

**FIGURE 6:**
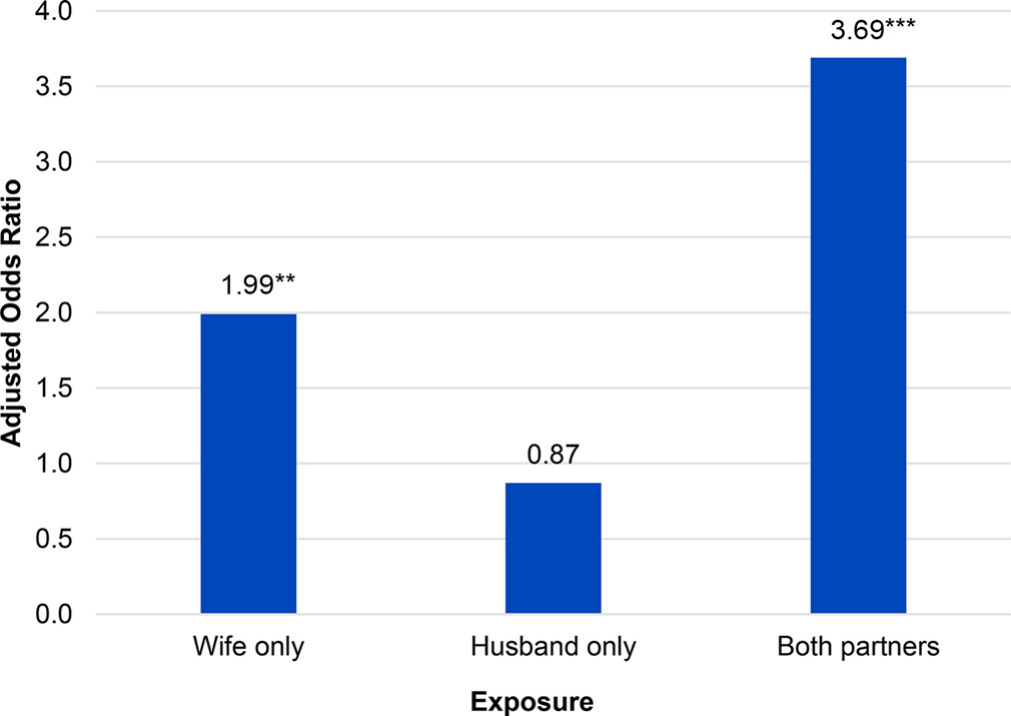
Adjusted Odds Ratios for Current Contraceptive Use Among Young Married Women Aged 15–24 With 1 Child, According to Exposure to PRACHAR Phase I Interventions (Wife, Husband, or Both) (N=1779) Reference group: neither partner exposed. Odds ratios adjusted for parity, education, and standard of living index. *P<.05; **P<.01; ***P<.001. Abbreviation: PRACHAR, Promoting Change in Reproductive Behavior of Adolescents.

Phase I results suggest that a gender synchronized approach in which both male and female partners are engaged—both together and separately—was associated with stronger results than working with only young men or only young women. Couples in which both the woman and her partner were exposed to PRACHAR had the highest odds of contraceptive use (aOR= 3.69; *P*<.001), whereas couples in which only the woman was exposed to PRACHAR had lower odds of contraceptive use (aOR=1.99; *P*<.01), and there was no significant effect on contraceptive use for couples in which only the husband was exposed (aOR=0.87; *P*>.05) (Figure 6). Similarly, Phase I and II data show that couples had higher odds of contraceptive use when wives participated in decision making about contraceptive use vs. when they did not participate (aOR=1.5 for couples without children and aOR=1.2 for couples with 1 child).

The PRACHAR data do not shed light on the effectiveness of the other PRACHAR intervention components. No significant associations were found between exposure to newlywed ceremonies or cultural programs and current use of contraception by young married women. The added impact of engaging mothers-in-law and other key gatekeepers could not be determined, as young married respondents were not asked if other family members besides their partner (i.e., parents, parents-in-law) participated in PRACHAR activities, and gatekeeper and community engagement to shift attitudes occurred across all Phase I and II intervention arms.

### Scalability and Effectiveness at Scale

As per an internal evaluation report by the Population Council, the scalable PRACHAR Phase III hybrid government-NGO model (using ASHAs instead of change agents and reducing or eliminating other activities) had significant but smaller contraceptive use gains than Phases I and II.^17^ From PRACHAR Phase III baseline to endline, the odds of currently using contraception increased 34% more among young married women in PRACHAR intervention areas than in comparison areas (aOR=1.34; *P*<.01, adjusted for age, education, and caste). Effect sizes varied by parity: aOR=1.89 among women with 1 child (*P*<.05), aOR=1.67 among women with more than 2 children (all *P*<.01), and no significant effect among women with zero or 2 children. The smaller effect sizes in Phase III may reflect several implementation factors. First, women in comparison and intervention areas had comparable rates of exposure to ASHAs (74% and 77%, respectively, in the 3 years prior to the survey). Second, among women contacted by ASHAs, only 28% of women in intervention areas and 17% of women in comparison areas reported that the ASHA discussed family planning. (For reference, 78% of women in intervention areas and 79% of women in comparison areas reported that the ASHA discussed child immunization.) Third, ASHAs had lower coverage rates for zero-parity women, which was a key target population for PRACHAR. Only 43% of zero-parity women were reached at least once in both intervention and comparison groups, compared with 78% to 86% of women of parity 1 or higher. Only 44% of zero-parity women visited by ASHAs received 13 or more visits in the past 3 years, compared with 61% to 74% of women of parity 1 or higher. Finally, Phase III also lacked significant behavior change interventions with gatekeepers such as mothers-in-law and other community influencers.

The hybrid government-NGO model, while scalable, had significant but smaller contraceptive use gains than the NGO-led model.

### Sustained Project Impact

**FIGURE 7:**
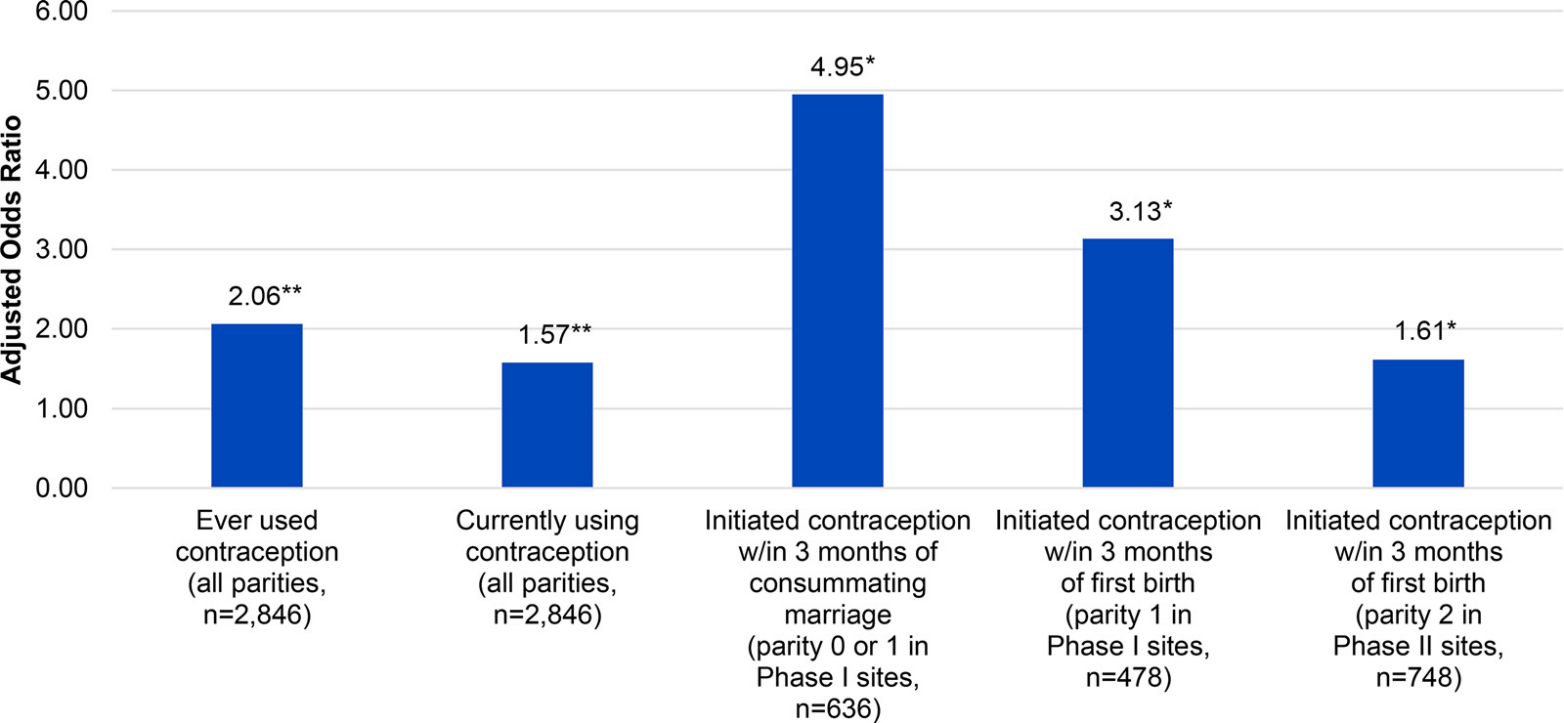
Adjusted Odds Ratios for Current Contraceptive Use Among Married Women Aged 15-34 in Areas Where PRACHAR Phase I, Phase II, or Phases I + II Were Implemented 4-8 Years Earlier Reference group is women in comparison areas where PRACHAR was not implemented. Adjusted odds ratios are from multivariate logistic regressions comparing baseline-endline changes in intervention and comparison areas. Odds ratios are adjusted for age, education, caste, and wealth quintile. *P<.05; **P<.01; ***P<.001. Abbreviation: PRACHAR, Promoting Change in Reproductive Behavior of Adolescents.

**FIGURE 8:**
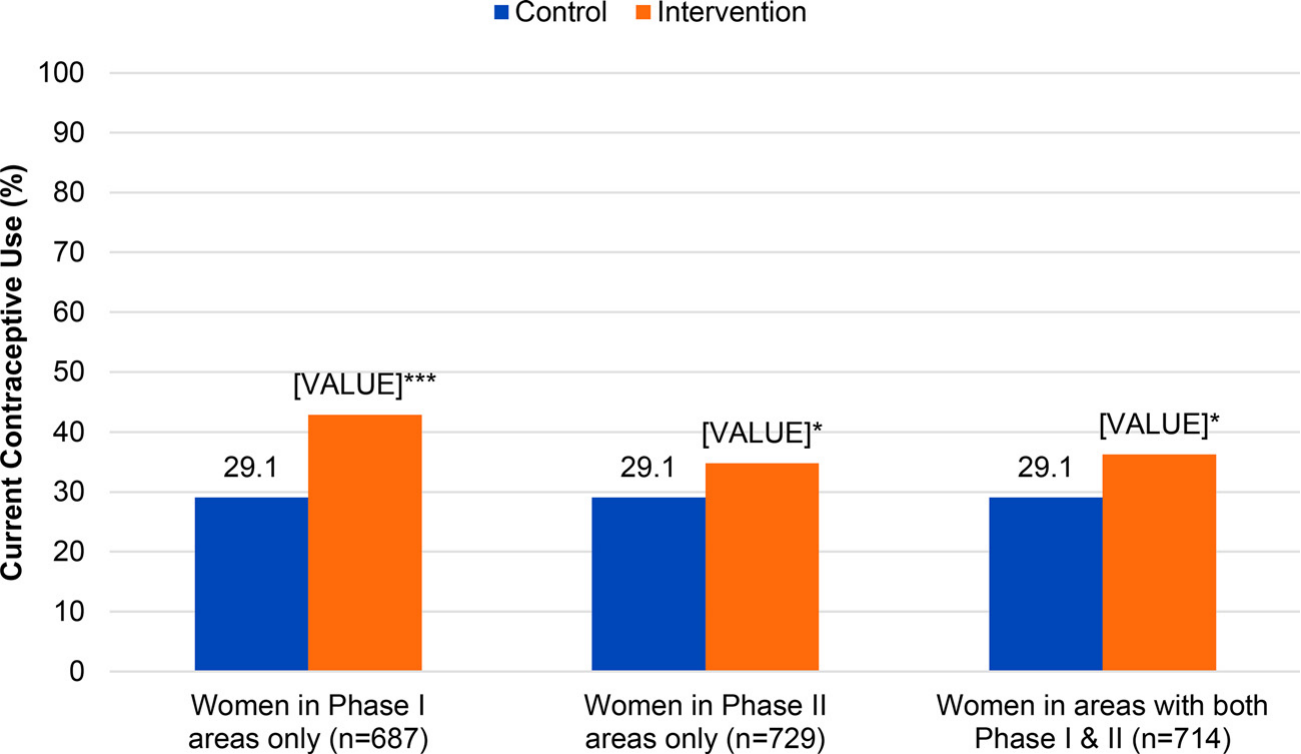
Current Contraceptive Use Among Married Women Aged 15–34 in Areas Where PRACHAR Phase I, Phase II, or Phases I + II Were Implemented 4–8 Years Earlier *P<.05; **P<.01; ***P<.001. Abbreviation: PRACHAR, Promoting Change in Reproductive Behavior of Adolescents.

As published in Jejeebhoy et. al (2015), the current contraceptive use gains achieved in PRACHAR Phases I and II persisted several years after the interventions ended, both among those directly exposed to PRACHAR as well as those living in the intervention areas at the time of the survey but not directly exposed to PRACHAR.^20^ Married women aged 15–34 in areas where Phases I and/or II were implemented 4–8 years earlier had 2 times higher odds of ever using contraception (aOR=2.06) and 57% higher odds of currently using contraception (aOR=1.57) than women in comparison areas where PRACHAR was not implemented (both *P*<.001) (Figure 7). Bivariate findings showed the highest current contraceptive use of 43% among married women living in areas where PRACHAR Phase I was implemented (Figure 8). Sustained effects were also seen for initiation of contraceptive use immediately after marriage and after first birth among specific parity groups. Married women with zero or 1 child living in former PRACHAR Phase I areas had nearly 5 times higher odds of initiating contraceptive use within 3 months of consummating marriage than women in comparison areas (aOR=4.95; *P*<.05), with 4.1% of women with zero children and 5.9% of women with 1 child initiating contraception in this time frame (vs. 0% and 2.1% in comparison groups, respectively). Married women with 1 child in former Phase I areas had 3 times higher odds of initiating contraceptive use within 3 months of their first birth (aOR=3.13), with 10.2% of women with 1 child initiating contraception during this time frame (vs. 2.9% in comparison areas). Married women with 2 children in former PRACHAR Phase II areas had 61% higher odds of initiating contraception within 3 months of their first birth (aOR=1.61) than women in comparison areas (both *P*<.05), with 15.1% of women with 2 children initiating contraception during this time frame (vs. 9.8% in comparison areas).

The contraceptive use gains achieved under the NGO-led PRACHAR model persisted several years after the interventions ended.

In addition to the contraceptive behavior changes sustained after the intervention ended, there is some evidence that PRACHAR led to sustained attitudinal shifts around healthy timing and spacing of pregnancies. Women in former PRACHAR intervention areas had significantly greater odds of preferring an ideal age at first birth of 21 or older (aOR=1.60) and preferring a birth interval of at least 36 months (aOR=1.46) than women in comparison areas (both *P<*.001).^20^ Several years after Phase I and II ended, qualitative data from program participants indicated that PRACHAR had played a role in shifting community perceptions on girls' sexual and reproductive health, specifically the use of contraception by adolescents and youth for healthy timing and spacing of pregnancies.^18^

## DISCUSSION

The evidence and learning generated from a decade of PRACHAR implementation have important implications for the design of future programming both in India and in a range of other contexts that face similar challenges of early marriage, early and rapid repeat pregnancies among young married women, and inequitable social and gender norms. The evidence and learning also raise critical questions around scale up and sustainability that should be explored in future programming for married youth.

### Comprehensive Programming Can Increase Contraceptive Use Among Married Young People

PRACHAR Phase I results demonstrate that a comprehensive program with multiple reinforcing interventions tailored to specific life stages and aimed at different levels of a socioecological model can effectively increase contraceptive use among married young people in a conservative context.

PRACHAR evaluations and implementation experience also demonstrate the importance of several other design features, particularly gender synchronization and the life cycle approach for adolescents and youth. When both young married women and their male partners were exposed to project interventions, contraceptive use was greater. This quantitatively demonstrates what the literature has long suggested—that changing behaviors related to contraception and fertility requires engagement of both members of the couple.^22^ While PRACHAR effectively engaged both members of the couple, the gender content of the intervention was somewhat limited, focusing primarily on enhancing young women's participation in household decision making. The social and behavior change interventions at the community level focused on norms related to marriage, contraception, and fertility, rather than underlying inequitable gender norms. As gender inequality is a primary driver of early marriage and early and rapid repeat childbearing among young women, future programs aiming to increase contraceptive use among young married women should more robustly address the gender inequitable attitudes, behaviors, and norms underlying contraceptive behavior and the intersectional vulnerabilities, such as poverty and lack of educational opportunities, that young married women face.

When both young married women and their male partners were exposed to project interventions, contraceptive use was greater.

PRACHAR included interventions that targeted adolescents and youth at different life stages, including before marriage, right after marriage, and before and after childbirth. The findings from the monitoring and evaluation data suggest that this approach—intervening at multiple points along the life cycle—had powerful impacts on the uptake of contraception. Young women who received home visits at multiple life stages as per the intervention design had the highest use of contraception, and women who had used contraception before their first birth were more likely to use contraception after their first birth than those who had not previously used contraception. This finding has relevance to a range of contexts and quantitatively reinforces the importance of interventions across the life cycle, including prior to the first birth.^23^

### Shifting From NGO to Government Workers Enhances Scalability but May Have Reduced Effectiveness

Though Phase I and Phase III evaluations did not use the same methodology so caution should be taken in direct comparison, the smaller magnitude of effect of PRACHAR Phase III (the government-NGO hybrid model) relative to the effect size of Phase I (the full comprehensive model) raises important considerations for program design and scale up.

First, the shift from NGO change agent (in Phases I and II) to ASHA (in Phase III) enhanced scalability. ASHAs are a scalable platform through which interventions can potentially reach many people and be sustained through government funding, whereas local NGOs have limited geographic reach and depend on external funding sources. However, the shift to ASHAs resulted in reduced quality and reach of interpersonal communication during home visits under Phase III. There are several possible reasons for this. Like community health workers in many other countries, ASHAs are responsible for promoting a wide range of maternal and child health care practices and are regularly tasked with additional priorities. During PRACHAR implementation, incentivization schemes that encouraged ASHAs to seek out pregnant women and refer them for institutional delivery diverted ASHAs from reaching young nulliparous women and inadvertently disincentivized discussion of family planning. Some states in India have explored incentivization of community health workers for helping couples to achieve outcomes related to delaying and spacing births, but ASHAs—like community health workers worldwide—remain a cadre with many responsibilities and competing incentives covering the gamut of maternal and child health. The PRACHAR experience under Phase III demonstrates the challenges of relying on government-supported multipurpose community health workers to conduct intensive behavior change focused on young married women, amidst their other competing priorities.

In addition, PRACHAR further demonstrates that shifting the main intervention to government implementation resulted in less funding and diminished prioritization of the other elements of the intervention, including reduced engagement of male partners and key gatekeepers. Considering the quantitative and qualitative evidence from Phases I and II of PRACHAR that points to the importance of the engagement of male partners and the broader community, it is possible that the reduced engagement of men and limited community social norm change interventions under Phase III contributed to the reduced magnitude of impact. This finding aligns with behavior change theory and reinforces existing evidence from a range of contexts that suggests that the use of a socioecological framework with multiple reinforcing interventions at different levels can change behavior related to contraceptive use among young married couples.^6^^,^^7^

The PRACHAR experience suggests that there are trade-offs for behavior change approaches when moving from a more intensive, NGO-implemented approach to an approach that may be more easily scaled but relies on overburdened government workers or systems. These trade-offs are not unique to PRACHAR and are likely to be found when seeking to scale up behavior change programs across a range of target groups and outcomes.^24^ Behavior change often requires moreintensive interpersonal and community-level efforts, which do not fit naturally into the mandate and scope of most government community workers or existing government systems, such as health facilities. For example, in India as in many other countries, there are no clear government workers or systems to take up interpersonal behavior change efforts with adolescents and youth and community-wide social and behavior change activities, which poses further challenges to scaling up through government-only systems. The Rashtriya Kishor Swasthya Karyakram (RKSK) program in India, launched in 2014, is the current national initiative to promote the health of adolescents and youth through interpersonal communication strategies, health services, and several other school- and community-based channels.^25^ However, RKSK uses unpaid youth peer educators, rather than a formal government system or cadre, as the primary implementer of the program component that aims to reach young people with interpersonal communication to catalyze behavior change. Governments, donors, and practitioners must think critically and creatively about the appropriate scale-up pathways for behavior change interventions that rely on interpersonal communication and analyze potential trade-offs between quality, impact, and scale. These considerations also suggest that practitioners could explore and rigorously evaluate methodologies for behavior change that require less intensive interpersonal and community-level interventions.

### Comprehensive Programming Can Have Sustained Effects on Behaviors

The PRACHAR experience also raises important questions on potential tensions between less intensive implementation approaches that can reach more people in the context of constrained funding but may have diminished impact on intractable behaviors, and intervention models that may be more intensive but catalyze change in behaviors with long-lasting and intergenerational effects. The PRACHAR long-term studies indicate that the more intensive interventions of PRACHAR Phases I and II had sustained impacts for 4 to 8 years after the program ended. While the PRACHAR studies did not include normative measures, the sustained behavior change and corresponding attitudinal changes regarding healthy timing and spacing of pregnancies suggest that norms surrounding use of contraception and fertility among young married couples may also have shifted due to PRACHAR. While direct comparison of the sustained impact of Phases I and II with Phase III is not possible (because no long-term study has been done following Phase III), it is important to consider whether the intensity and/or quality of Phase I and II interventions might have contributed to the sustained effects observed. Would we see the same sustained effects from the less intensive ASHA-led intervention with limited community engagement, especially given that the immediate effects on contraceptive use at endline were modest (and negligible among zero-parity women)? We hypothesize that we would not. This begs the question of whether a more intensive model that creates sustained impacts after 3 years of implementation may offer better value for money than a less intensive model that reaches more people but must continue for many more years to create long-lasting effects.

## CONCLUSION

PRACHAR represents a decade of investment in implementation, learning, and evaluation of social and behavior change efforts to increase voluntary contraceptive use for married adolescents and youth. It is essential that we use the learning from PRACHAR to inform the design and implementation of current and future programs for married youth. In addition, to advance the field and better meet the needs and rights of married adolescents and youth, future programs should seek to answer the questions that PRACHAR raises in relation to effective models to scale up multilevel behavior change efforts and around potential trade-offs between intensity, quality, and sustainability of impacts over time. Answering these questions will require implementation of thoughtfully designed programs for married youth that draw on the learnings from PRACHAR and improve upon the PRACHAR model. In addition, it will require robust and mixed-method evaluations and thoughtful implementation science approaches that assess not only effectiveness but also how programs are implemented, why programs are or are not impactful, and what the scale-up pathways and processes are. Furthermore, as the use of social norm measurement and theory advances in global public health, programs for married youth will benefit from more theory-driven normative change interventions and measurement.^26^

## Supplementary Material

17-00440-Subramanian-Supplement.pdf

## Notes

**Peer Reviewed**

**Funding:** The Bill & Melinda Gates Foundation funded the Momentum Project to disseminate learnings from PRACHAR to inform contraceptive programming with married youth. The David and Lucile Packard Foundation funded all 3 phases of the PRACHAR Project and the program learning grant to synthesize lessons learned from PRACHAR. UNFPA funded a portion of PRACHAR Phase III implementation.
