# Supplementary material for: Increasing Contraceptive Use Among Young Married Couples in Bihar, India: Evidence From a Decade of Implementation of the PRACHAR Project
Source: Glob Health Sci Pract. 2018 Jun 27;6(2):330–44. doi: 10.9745/GHSP-D-17-00440 (PMC6024625; doi:10.9745/GHSP-D-17-00440)
Supplement: 17-00440-Subramanian-Supplement.pdf [file 17-00440-Subramanian-Supplement.pdf]

Subramanian L, Simon C, Daniel EE. Learning from a decade of the PRACHAR Project in Bihar, India: evidence and implications for programming to increase contraceptive use among young married couples. *Glob Health Sci Pract*. 2018;6(2). https://doi.org/10.9745/GHSP-D-17-00440

SUPPLEMENT. How PRACHAR interventions were implemented with young married couples, key influencers, and communities

This supplement contains further details on the implementation of the key Promoting Change in Reproductive Behavior of Adolescents (PRACHAR) interventions. Please note that this supplement does not describe the activities that were specifically for unmarried adolescents, as this paper focuses on the young married couples and the interventions with their key influencers.

Counseling through home visits to married young women with no children, married young women with first pregnancy, married postpartum young women who delivered their first child, married young women with one child

The primary intervention used to reach young married women was one-on-one counseling conducted via home visits. In Phase I and in the comprehensive arms of Phase II, the home visits were conducted by change agents who were selected and supported by local NGO implementing partners.

The selection criteria for the change agents including the following requirements: have 10 years of schooling, be willing to devote the required time to project activities, be willing and able to discuss family planning/reproductive health (FP/RH) issues with sensitivity and cultural relevance, and be able to go out to visit households. The local partner NGO staff talked to large groups of people from the village to announce the recruitment process and selection criteria, and the project staff then interviewed potential candidates to finalize the selection. Village leaders did not participate in the selection process because the project wanted to avoid patronage and promote transparency and effectiveness. The project selected 1 female change agent per village. Male change agents (1 male change agent per approximately every 3 villages) were recruited for the group-based activities discussed. Each female change agent was responsible for conducting home visits with 100–125 young married women and received small compensation (approx. 80 rupees per month at the time of implementation). Most of the female change agents had this as their only job (they were previously only working in the home), and many became Accredited Social Health Activists (ASHAs) afterward.

The change agents received 7 days of training annually, for a total of approximately 21–24 days of training in Phase 1 and the comprehensive arms of Phase II. The first training covered the PRACHAR project, contraception (including issues such as whether the husband has to give permission for a young woman to use contraception), and counseling skills on contraception, with a focus on rights, attitudes, and behaviors that the project was seeking to increase. The training focused on building change agents' confidence and acceptance/positive attitudes toward contraception and FP/RH for young couples, so they could be effective in changing other people's attitudes and behaviors. The training also covered how to identify the project target groups, develop beneficiary listings, and use flipbooks for different cohorts. After the first training, the change agents were trained on additional topics at annual intervals. Change agents were supervised by the local NGO partners, with back-up support and frequent monitoring from Pathfinder International staff.

A detailed schedule of home visits was developed for each target audience depending on their life cycle stage (newlyweds with no children; pregnant with first child; first-time mothers immediately postpartum; and first-time mothers with young children), with timing and frequency of visits determined according to the desired outcome for each group. The overall strategy was to provide focused, relevant information to people when they most needed it, in order to avoid information overload and enhance use of information to change behavior.

The home visit schedule was as follows:

- Newlyweds: change agents conducted 2 visits in the first month to establish rapport with the newlywed woman and mother-in-law and promote the importance of delaying first birth. After that, change agents conducted monthly follow-up visits to support adoption and continuation of contraception, including support for correct/consistent use of the selected contraceptive methods (usually the pill and condoms, given service delivery and policy constraints at the time).
- Pregnant women: typically received at least 3 home visits—1 visit during the first 3 months, the second between the 4th and 6th month of pregnancy, and the third before she left her marital home to go to her maternal home for the delivery. Pregnant women received advice on antenatal care and safe delivery, were educated on postpartum care (including exclusive breastfeeding, weaning, immunization, child nutrition, and contraceptive use to space subsequent births), and were referred for health services.
- Immediate postpartum women: the WHO home visit protocol at the time was used (1st day, day 7, day 14, etc.) but extended up to day 45. Visits focused on danger signs for mother and baby and spacing the next child, with encouragement to adopt a modern contraceptive method within 45 days postpartum.
- Women with 1 child or more: monthly home visits to support use of contraception to space pregnancies, until the woman became pregnant again (in which case the protocol for pregnancy would take place).

Phase II tested several variations on the change agent-led home visit mode. These included:

- Volunteer-only model: In this arm of Phase II, the team sought to understand if volunteers could take the place of paid change agents. Volunteers were selected from communities. Many had their own occupations, so dedicated only some time to PRACHAR. Volunteers were not able to follow the full visit protocols noted above given time constraints, so they did as much outreach as time would allow. Volunteers received a 3-day training. Female volunteers were encouraged to provide counseling on contraception and FP/RH to young married women, and male volunteers were encouraged to reach men in the household.
- Trained couples-only model: In this arm of Phase II, married couples were trained to take the place of change agents. A one-time 3-day training on FP/RH was conducted separately for married men and women (0 and 1 parity). Young couples were encouraged to conduct peer outreach to other young married men and women in their community and provide information on FP/RH.

Phase III home visit: Under Phase III of PRACHAR, home visits to young married women were conducted by the government ASHAs rather than the paid NGO-supported change agents. Under Phase III, Pathfinder worked closely with key stakeholders, including the Government of Bihar, UNFPA, and other training agencies in the state to review and revise the PRACHAR Female Change Agent training manual to create new ASHA training modules. The existing ASHAs were identified by the government for training on the PRACHAR approach. Training was conducted by the government and local partners and supervised by Pathfinder staff. The ASHA training for the PRACHAR approach was a 4-day, nonresidential training program. ASHAs were trained on effective communication of FP/RH information to be delivered through home visits, focusing on behavior change for delaying first births, spacing between the first and second births, and limiting births by those who have achieved their desired family size. After the training, each ASHA was expected to conduct home visits every month to motivate young couples to use contraception to delay and space their pregnancies. The ASHAs submitted home visit reports to the NGO implementing partner supervisor every month, and this time was used for any clarifications and support needed. The ASHAs were formally supervised through the government structure.

Newlywed couple ceremony/infotainment party

This intervention was conducted in Phase I only. A high priority was placed on immediate intervention with newlywed couples to promote their ability to make informed choices about the use of contraceptives to delay the first child. The *Nav Dampati Swagat Samaroh* (NDSS) ("Newly-Married Couples' Welcome Ceremony) involved short plays, games, and couple activities to discuss FP/RH, the elements of happy family life, the economics of raising children, and joint decision making. These activities were conducted by the local implementing partners on a regular basis. Couples were given cooperative tasks to perform together in order to practice communication and shared decision making. Skits illustrated how to negotiate with parents or in-laws who pressure them on childbearing. Finally, couples received a small lunchbox containing condoms and combined oral contraceptive pills to encourage immediate adoption of contraception. Careful explanations of the different kinds of contraceptives and how they are used were initiated during these ceremonies and followed-up in the coming weeks through home visits. In order for young recently married women to attend the NDSS events, it was essential to enlist the cooperation and support of the mothers-in-law, which was done through meetings with change agents.

Group discussions and dialogues led by change agents

The PRACHAR approach included a number of different small-group activities targeting different populations. Small-group meetings/activities were held, separately, with newly married husbands or husbands with young wives, fathers-in- law, mothers-in-law, and young married women (usually separated by parity). Each local partner worked with the change agents to develop detailed quarterly schedules for each group meeting.

Male change agents (Phase I and II)/male communicators (Phase III) led the group meetings for husbands and fathers-in-law, and female change agents led the groups for mothers-in-law and young

married women. Small-group participants generally attended 3 group meetings of approximately 2 hours each. Each group had a maximum of 10–15 people, and the groups were held locally where people lived. The group meetings for parents-in-law were intended to sensitize family influencers and encourage their support for the contraceptive behavior of their children.

Topics varied based on group participants, and change agents used story-based flipbooks to guide conversation and discussion, based on 2 storytelling modules: *Hariyala Banna* (pictorial short story highlighting the concept of delaying marriage) and *Banno ki Shidi* (pictorial short story highlighting the concept of delaying first birth and spacing the second birth). In addition to general FP/RH information, including the benefits of delaying the first birth and spacing subsequent births, participants discussed the economic and health benefits of delaying childbearing. Groups also dialogued on household dynamics and norms around gender roles and household decision making.

Community engagement

PRACHAR used a number of different techniques to create an enabling environment for young couples to use contraception to delay and space pregnancies. The techniques included:

- Wall paintings (Phase I and II): PRACHAR developed murals a village level depicting key behaviors desired by the project, including couples talking about the importance of young couples delaying and spacing pregnancies.
- Street theater (Phase 1 only): Local NGOs with specialization in street theater were responsible for conducting street theater at village level. The local NGOs were rigorous in their preparation and execution of the street theater in order to make them realistic and inspire discussion and the challenging of community norms. The street theatre performers were selected rigorously and trained in the principles of street theater. A 4-day script development workshop was followed by 15-day performing skills workshop and refresher trainings. Performers also underwent a 4-day training program on contraception to enable them to handle commonly asked questions from audiences. There was 1 street play every 3 months in every village. Actors were paid per performance.
- Orientation and training of community leaders and influencers on RH for young people (Phase I and Phase II comprehensive model only): PRACHAR sensitized key leaders and influencers in communities in order for them to proactively support program implementation, encourage behavior change in the community, and facilitate to overcome implementation barriers. The project team identified elected community representatives of Panchayati Raj Institution, community social workers, respected elderly members of the community, volunteer school teachers, and local leaders recognized by the community. PRACHAR provided 2 days of orientation training for these leaders/influencers on programmatic issues such as need, importance and scope of delaying marriage, first birth and spacing the second and subsequent births, as well as on the key program approaches, strategies, and activities to be implemented in the community along with the structure of the team of program functionaries and their roles in the project.

Service delivery improvement and linkages

Phase I and II included the following interventions to link young people with services:

- Monthly maternal and child health (MCH) clinics: Pathfinder and local partners worked with the government to ensure that the government auxiliary nurse-midwives participated in monthly MCH clinics. Though much of the focus of the MCH clinics remained on immunization, there was an increase in the provision of antenatal care and postpartum care, family planning counseling, and basic health checks for adolescents (who were invited to the clinics after their training programs). MCH clinics were provided a kit of basic instruments (weighing scale, blood pressure apparatus, stethoscope, and fetoscope).
- The project trained Traditional Birth Attendants and Rural Medical Practitioners in birth preparedness and basic obstetric care, harm reduction, recognition/referral of obstetric emergencies, and postpartum contraception. Project staff held periodic meetings with trainees to reinforce knowledge/skills.
- Increase availability of contraceptives: Local pharmacy owners/shopkeepers were trained and encouraged to procure nonclinical contraceptives from social marketing agencies and sell them through their retail outlets.
- Referral linkages with public and private providers: To enable referral of cases, the project surveyed and listed qualified providers of reproductive health services in the intervention blocks and districts and developed a list that could be used for referral purposes. The project also monitored stock availability of pills and condoms at local social marketing outlets and alerted the responsible social marketing agency when stock-outs were noted.

NGO capacity building

PRACHAR was implemented in partnership with a large number of local NGOs. In Phase I, PRACHAR worked with 19 implementing NGOs and 12 training NGO. In Phase II, PRACHAR worked with 10 implementing NGOs and 9 training partners, and with 9 implementing partners and 2 training partners in Phase III. PRACHAR included robust capacity building efforts to ensure local partners developed the sustainable capacity to design, implement, and monitor FP/RH programs for young people. Each Pathfinder PRACHAR project officer worked with 3–4 NGOs, visiting them on a weekly basis to closely monitor performance and provide guidance and mentorship to NGO staff.

The Pathfinder PRACHAR project officers conducted assessments of each NGO and worked with the NGOs to build skills and capacities in areas noted as lacking in the assessments through both classroom-based training and practical field experience. Capacity building included technical topics, such as sexual and reproductive health for adolescents and youth and gender; development of performance and progress monitoring systems; strengthening of accounting and financial systems; project planning and implementation techniques; behavior and norm change principles; and the use of monitoring data for decision making.
